# The Characterization and the Biological Activity of Phytotoxin Produced by *Paraphoma radicina*

**DOI:** 10.3390/jof8080867

**Published:** 2022-08-17

**Authors:** Shu-Zhong Dang, Yan-Zhong Li

**Affiliations:** 1 Key Laboratory of Grassland Livestock Industry Innovation, Ministry of Agriculture and Rural Affairs, Lanzhou 730020, China; 2 Engineering Research Center of Grassland Industry, Ministry of Education, Lanzhou 730020, China; 3 College of Pastoral Agriculture Science and Technology, Lanzhou University, Lanzhou 730020, China

**Keywords:** *Paraphoma radicina*, phytotoxin, pathogenic activities, alfalfa root rot

## Abstract

*Paraphoma radicina* is a new pathogen that causes alfalfa paraphoma root rot (APRR), leading to alfalfa production losses. The resistance levels of 30 alfalfa cultivars to APRR have already been characterized. However, the pathogenic mechanism of *P. radicina* is still unclear. This study aimed to assess the effects of a crude toxin extracted from *P. radicina* cell-free culture filtrate (CFCF) on susceptible and resistant cultivars of alfalfa. Meanwhile, the crude toxin components were detected using gas chromatography-mass spectrometry (GC-MS) analysis. CFCF cultured in MEB medium for 14 days and crude toxin extracted by ethyl acetate induced significant phytotoxicity caused the average lesion areas of 5.8 and 3.9 mm^2^, respectively, on alfalfa leaves. The crude toxin exhibited resistance to high temperature, as shown by a lesion area of 3.6 mm^2^ when treated at 120 °C for 30 min. Different concentrations of the crude toxin in water and MS medium had different effects on susceptible and resistant cultivars. Moreover, the crude toxin affected the plasma membrane, mitochondria, and nuclear membranes of alfalfa root cortical cells. Further, it induced significant phytotoxicity on *Sonchus oleraceus* L., *Capsella bursa-pastoris* (Linn.) Medic, and *Chenopodium album* L. *Agropyron cristatum* L. (average lesion areas; 11.6, 15.8, 21.4, and 6.2 mm^2^, respectively), indicating that the crude toxin of *P. radicina* is a non-host-selective toxin. GC-MS analysis detected four possible active substances in the toxin (3-hydroxypyridine, 5-methylresorcinol, 3-Hydroxypropionic acid, and 4-Hydroxyphenylethanol). Therefore, this study may provide insight into the pathogenic mechanism of *P. radicina* to alfalfa.

## 1. Introduction

Alfalfa is the most cultivated forage in the world because of its high economic and ecological benefits. Alfalfa root rot, an important disease affecting the yield and quality of alfalfa, is globally widespread. *Paraphoma radicina* is a new pathogen reported to cause alfalfa root rot [1]. The taxon *Paraphoma* was initially a section of the genus *Phoma* but is currently classified as a distinct genus due to its characteristic of setose pycnidia and dictyochlamydospores [2]. *P. radicina* is the typical *Paraphoma* species that was first isolated from *Prunus cerasus* roots [3]. Alfalfa paraphoma root rot (APRR) was the first known disease caused by *P. radicina*. APRR is characterized by root discoloration, necrosis, and stunted growth during the re-greening period [4]. 

In the process of pathogens infecting the host, fungal phytotoxins play an important role [5]. Phytotoxins are microbe-produced secondary metabolites and are important factors that could facilitate pathogen colonization of host tissues [6]. Some phytotoxins can directly kill cells, thus enhancing infection of dead cells. Other phytotoxins interfere with the induction of defense responses, generating necrosis required for pathogenesis [7]. Phytotoxin causes plant wilting and reduced growth, chloroses, necroses, and spotting of aerial parts of plants [8]. The fungal phytotoxins can produce some or even all the symptoms produced by pathogens [9,10]. Several past studies have reported the diverse effects of phytotoxins. For instance, perylenequinone toxins (produced by *Cercospora* sp., *Alternaria alternata*, *Cladosporium*) affect plant photosystem II and increase the content of reactive oxygen species in the cell, thus increasing membrane permeability [11]. Fusaric acid (FSA) phytotoxin (produced by *Fusarium oxysporum*) impairs the respiration activities in mitochondria and damages the host cell membranes, leading to water loss in leaves [12,13]. Many phytotoxins inhibit plant enzyme activity and disrupt the biosynthesis of critical metabolites. For example, AAL toxin (produced by *Alternaria alternata* f. sp. *Lycopersici*) inhibits L-aspartate activity [14].

Fungal phytotoxins have been widely used to develop resistant cultivars [15,16,17]. They have also been used as primary compounds or templates for the development of new synthetic herbicides [18,19]. Pyrenophorin phytotoxins (produced by *Dreschlera avenae*) are efficient against wild oats (*Avena sterilis* L.) [20]. A previous study found that fusicoccin and its derivatives have activities against the parasitic weed striga (*Striga hermontica*) [21]. *Phoma*-like fungi cause severe diseases in many plant species. Moreover, some *Phoma*-like fungi can produce phytotoxin secondary metabolites. Numerous studies have indicated that phytotoxins produced by *Phoma*-like species promote pathogen infection [22,23,24]. For instance, two phytotoxin compounds herbarumins I and II, isolated from *Phoma herbarum*, can significantly inhibit radicle growth of *Amaranthus hypochondriacus* seedlings [25]. Two main phytotoxins from *Phoma asparagi* (altiloxins A and B) can cause stem blight disease on asparagus. The two compounds can also inhibit the root elongation of lettuce seedlings [26]. Moreover, two phytotoxin compounds have been isolated from *Paraphoma* sp. (phaeosphaeride A and B). Phaeosphaeride A has herbicidal activity [27].

This study aimed to assess the biological activities of a crude toxin from *P. radicina* on susceptible and resistant cultivars of alfalfa. To the best of our knowledge, this is the first study to investigate the effect of crude toxin from *P. radicina*, on alfalfa. Therefore, this study may provide insights into the pathogenic mechanisms of *P. radicina* in alfalfa. 

## 2. Materials and Methods

### 2.1. Fungal Cultures and Cell-Free Culture Filtrate (CFCF) Production

*P. radicina* strain LYZ0187 was cultured on oatmeal agar (OA) medium and kept in the dark at 25 °C for 1 mon. Six mycelial plugs from OA cultures (diameter: 5 mm) were seeded in 1000 mL Erlenmeyer flasks containing 400 mL of potato dextrose broth (PDB), oatmeal both (OB), and malt extract broth (MEB). The cultures were then incubated in the dark at 25 °C on a rotary shaker (150 rpm) for 7, 14, and 21 days. The sample was passed through four layers of cheesecloth and centrifuged at 5000× *g* for 20 min to obtain culture filtrates. The pellet was removed, and the supernatant was filtered in a vacuum via microfiltration using 0.45 μm sterile microfilters to obtain CFCF. The CFCF was stored at 4 °C. A leaf necrosis assay was conducted to detect the toxicity of the CFCF. An MEB culture filtrate was used for all subsequent tests unless stated otherwise.

### 2.2. Extraction of Crude Toxin from the Pathogen Culture

Crude toxin was extracted [28] with minor modifications. Briefly, 50 mL of CFCF was put in a separating funnel and then the same volumes of petroleum ether, chloroform, diethyl ether, and ethyl acetate were added, respectively. The contents were then shaken, then kept static until the two phases separated. The process was repeated thrice. The organic fractions were combined and evaporated in a vacuum desiccator to dryness to obtain crude toxin. The different organic solvents extracted from the crude toxin were named organic solvent, viz. petroleum ether, chloroform, diethyl ether, and ethyl acetate. The crude toxin was dissolved in sterile water (5 mL) for toxicity analysis using leaf necrosis assay. Sterile water and untreated culture filtrate were used as the control. Unless otherwise stated, the crude toxin extracted by ethyl acetate was used for all subsequent tests.

### 2.3. Effect of Heat Treatment on Stability of Crude Toxin Activity

The crude toxin was subjected to 60, 80, and 100 °C in a water bath and in 120 °C autoclaved for 30 min to determine the thermostability of phytotoxins. The biological activity of the crude toxin was measured using a detached leaf bioassay. Untreated crude toxin (25 °C) was used as the control. 

### 2.4. Detached Leaf Bioassay

A leaf necrosis assay was performed to determine the biological activities of culture filtrate and crude toxin (Gibraltar) [29]. Healthy leaves of the same sizes were selected for this analysis. The leaf surface was sterilized with 75% ethanol and four wounds were picked with a needle on each leaf. The leaves were placed on a sterile petri dish with a double layer of moist sterile filter paper and 5 μL of the CFCF or crude toxin was applied to the wound. The petri dishes were incubated at 25 °C for 3 days. The lesion area around each wound was then assessed. These treatments were conducted six times, and all bioassays were repeated at least thrice.

### 2.5. Effect of the Crude Toxin on Susceptible and Resistant Cultivars

#### 2.5.1. Effect of Crude Toxin in Water Medium

Seeds of susceptible and resistant cultivars were surface-sterilized using 75% ethanol for 1 min and 2% sodium hypochlorite for 8 min and then rinsed thrice using sterile water [30]. The seeds were planted in Petri dishes (diameter: 9 cm) with filter paper containing 5 mL different concentrations of crude toxin (5 × 10^−4^, 1 × 10^−3^, 2 × 10^−3^ g/mL). Each plate had 30 seeds. The Petri dishes were incubated in the dark at 25 °C for 3 days. The germination rate of the seeds was then determined. Plants in 5 mL 20% ethyl acetate were used as the control. Each treatment was repeated three times.

Radicle growth in susceptible and resistant cultivars in a water medium supplemented with the crude toxin was determined as follows, the seeds of resistant and susceptible cultivars were incubated in Petri dishes with moistened filter paper at room temperature (25 °C) for 36 h. The germinated seedlings (when the radicle length approach to seeds length considered as germination) were incubated in Petri dishes (diameter: 9 cm) with filter paper containing 5 mL of crude toxin with different concentrations (5 × 10^−4^, 1 × 10^−3^, 2 × 10^−3^ g/mL). Each plate had 15 seeds. The Petri dishes were incubated at 25 °C for 3 days (12 h photoperiod). The root length of the seedlings was then measured. Plants in 20% ethyl acetate were used as the control. Each treatment was repeated three times.

#### 2.5.2. Effect of Crude Toxin in MS Medium

The inhibitory effect of different concentrations of the crude toxin on seed germination and seedling growth in the susceptible and resistant cultivars was assessed through MS medium bioassay (5 × 10^−4^, 1 × 10^−3^, 2 × 10^−3^ g/mL). The crude toxin was added to each phytojar containing MS medium, and the medium were sterilized by autoclaving at 120 °C for 30 min. The surface-sterilized seeds were sown in phytojars, with 20 seeds per jar. The jars were then sealed with parafilm tape and put in a controlled environment at 22 ± 2 °C with a photoperiod of 12/12 h light/dark cycle for 3 days. MS medium with 20% ethyl acetate was used as the control. The germination rate of the susceptible and the resistant cultivar seeds was then determined. Each treatment was repeated three times.

The seedling growth in MS medium was determined as follows: The surface-sterilized seeds were pre-germinated for the assay at 22 ± 2 °C for 36 h; seeds were considered germinated when the radicle length was equal to the seed length and the germinated seeds were selected for the follow-up experiment. The MS medium with different concentrations of crude toxin was sterilized by autoclaving at 120 °C for 30 min. Fifteen pre-germinated seeds were sown in each phytojar, as described above. MS medium supplemented with 20% ethyl acetate was used as the control. The seedlings were harvested after 3 days, thoroughly washed with water, and the length of radicle and shoot were measured. Each treatment was repeated three times.

### 2.6. Transmission Electron Microscopy

The susceptible and the resistant cultivar seeds were placed in Petri dishes with a double layer of moist sterile filter paper. The Petri dishes were then incubated in the dark at 25 °C for 2 days, then transferred to 12 h photoperiod for 3 days. The seedlings were treated with a crude toxin (2 × 10^−3^ g/mL). The seedlings treated with 20% ethyl acetate were used as the control. The roots of the treated seedlings were then incubated at 25 °C for 3, 6, 12, 24, 48, and 72 h. The rhizome of the treated seedlings was cut into small pieces (shorter than 3 mm). The samples were fixed with 2.5% glutaraldehyde in phosphate buffer (PH 7.0) for 24 h, washed thrice using phosphate buffer, then post-fixed with 1% osmium tetroxide in phosphate buffer at 4 °C for 2 h. The sections were dehydrated with ethanol and embedded in resin. The samples were cut into extremely thin sections (70–90 nm) using a Lecia EM UC7 ultramicrotome fitted with a diamond knife. The sections were stained with 0.5% uranyl acetate and lead citrate, then visualized using a Tecnai G2 Spirit Bio-TWIN 120 KV (FEI, Hillsboro, American) electron microscope.

### 2.7. Host Selectivity Test

The common weeds in the alfalfa planting area (*Sonchus oleraceus* L., *Capsella bursa-pastoris* (Linn.) Medic, and *Chenopodium album* L. *Agropyron cristatum* L.) in China were selected to determine whether the crude toxin can induce phytotoxicity to other hosts. Three leaves of each weed plant were cleaned thrice using 75% ethanol, followed by a sterile deionized water washing. A leaf bioassay was then conducted as described earlier. A 20% ethyl acetate was used as the control. The lesion area around each wound was measured after 3 days.

### 2.8. Gas Chromatography-Mass Spectrometry

Pre-cold extraction mixture (400 μL) (methanol/chloroform (v:v) = 3:1) with internal standard (adonitol, 0.5 mg/mL stock) were added into a 2 mL tube containing crude toxin and vortexed for 30 s. A steel ball was then added to the sample and put in a grinding instrument (40 Hz) for 4 min. The sample thrice underwent ultrasonic treatment in an ice water bath for 5 min. The sample was centrifuged at 12,000 rpm (RCF = 13,800× *g*, R = 8.6 cm) at 4 °C for 15 min to obtain a supernatant. The supernatant (100 μL) was transferred to a fresh tube and evaporated in a vacuum concentrator. Methoxyamination hydrochloride (40 μL, 20 mg/mL in pyridine) was added to the sample, then incubated at 80 °C for 30 min. The sample was derivatized using 50 μL of BSTFA reagent (1% TMCS, *v*/*v*) at 70 °C for 1.5 h. The samples were gradually cooled to room temperature. All samples were analyzed using gas chromatography coupled with a time-of-flight mass spectrometer (GC-TOF-MS).

An Agilent 7890 gas chromatography coupled with a time-of-flight mass spectrometer was used for GC-TOF-MS analysis. The system utilized a DB-5 MS capillary column. The sample (1 μL) was injected in splitless mode. Helium was used as the carrier gas (front inlet purge flow rate; 3 mL min^−1^ and flow rate through the column: 1 mL min^−1^). The initial temperature was kept at 50 °C for 1 min, then increased to 310 °C (10 °C min^−1^) for 8 min. The injection, transfer line, and ion source temperatures were 280, 280, and 250 °C, respectively. The energy in electron impact mode was −70 eV. The mass spectrometry data were acquired in full-scan mode with the *m*/*z* range of 50–500 at a rate of 12.5 spectra per second after a solvent delay of 6.27 min. Raw data analysis, including peak extraction, baseline adjustment, deconvolution, alignment, and integration was conducted using Chroma TOF (V 4.3xLECO) software. A LECO-Fiehn Rtx5 database was used for metabolite identification by matching the mass spectrum and retention index. 

### 2.9. Data Analysis 

For all statistical analyses, SPSS 21 software was used. Two-way analysis of variance (ANOVA) was used to determine the differences in the two factors, culture media and incubation time, of toxin production by *P. radicina*. One-way analysis of variance (ANOVA) was used to determine the differences in seed germination rate, shoot, and radicle length and the inhibition rate of crude toxin on germination, shoot length, and radicle length. The least significant differences (LSD) were used to determine the differences between means (*p* ≤ 0.05).

## 3. Results

### 3.1. Toxin Production

**Figure 1:**
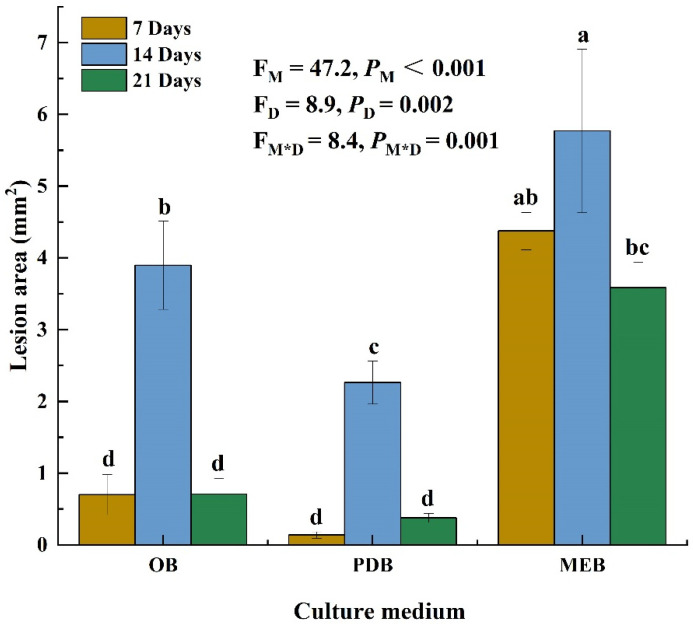
Phytotoxicity for cell-free culture filtrate (CFCF) produced by *Paraphoma radicina* that cultured in different medium and times. The mean lesion area was calculated from six leaves of tissue-cultured alfalfa plantlets (*n* = 6). Values (means + SE) marked with different letters in each column indicate significant differences (*p* < 0.05) based on one-way ANOVA followed by Duncan’s multiple range test. *P*_M_-values, *P*_D_-values, and *P*_M*D_-values of the ANOVA indicate significant differences *p* < 0.05 (independent *t*-test) culture medium (M), incubation days (D), and their interaction (M*D), respectively.

Three liquid mediums were used to examine the toxicity of *P. radicina* filtrates on alfalfa leaves to obtain the optimal liquid medium. The culture medium (*p* < 0.001), incubation time (*p* = 0.002), and the interaction between the two factors (*p* = 0.001) significantly affected the lesion area on the alfalfa leaves. Filtrates of *P. radicina* from 7-day OB, PDB, and MEB cultures resulted in lesions with areas of 0.7, 0.1, and 4.4 mm^2^, respectively. Moreover, the *P. radicina* filtrates from 14-day OB, PDB, and MEB cultures caused lesions with areas of 4, 0.4, and 5.8 mm^2^, respectively. Filtrates from 21-day OB, PDB, and MEB cultures produced lesions with areas of 0.7, 2.3, and 3.6 mm^2^, respectively (Figure 1). These findings indicate that filtrate from 14-day MEB cultures was significantly more toxic to alfalfa leaves than filtrates from other medium and time points. However, filtrates from non-inoculated medium caused no lesions on alfalfa leaves. Therefore, filtrate from the 14-day MEB culture was selected for subsequent tests.

### 3.2. Crude Toxin Extraction

**Figure 2:**
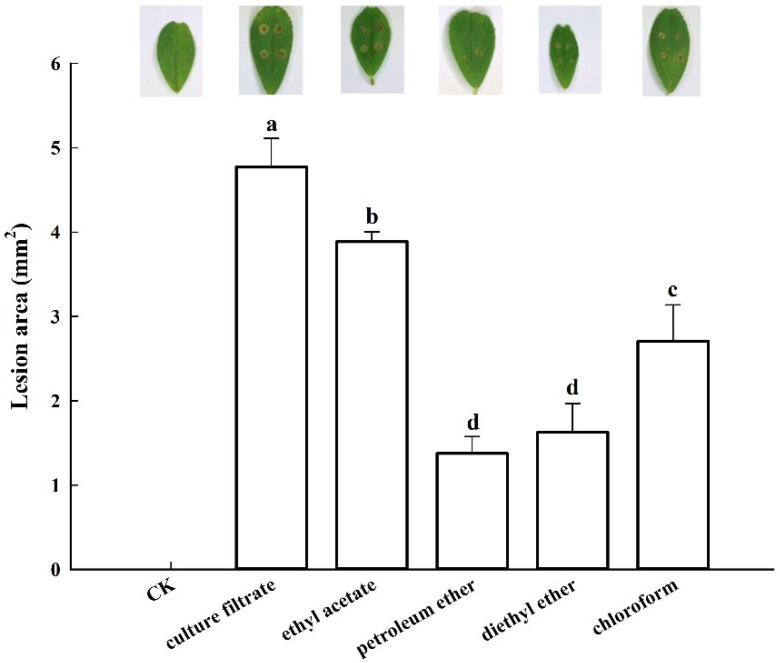
The crude toxin phytotoxicity of solvent extraction by different organic. The mean lesion area was calculated from six leaves of tissue-cultured alfalfa plantlets (*n* = 6). Values (means + SE) marked with different letters in each column indicate significant differences (*p* < 0.05) based on one-way ANOVA followed by Duncan’s multiple range test.

The filtrates of MEB were extracted using ethyl acetate, petroleum ether, diethyl ether, and chloroform. Toxins were detected in all solvent fractions (Figure 2) as sunken spots of irregular sizes. The solvent fractions of ethyl acetate, petroleum ether, diethyl ether, and chloroform resulted in lesions with areas of 3.9, 1.4, 1.6, and 2.7 mm^2^, respectively. The control group (non-inoculated medium) did not form a lesion. The filtrate of MEB culture caused a lesion with an area of 4.8 mm^2^. Ethyl acetate extract showed significantly higher toxicity than other organic solvents (*p* < 0.05) and was thus used for extraction in subsequent tests.

**Figure 3:**
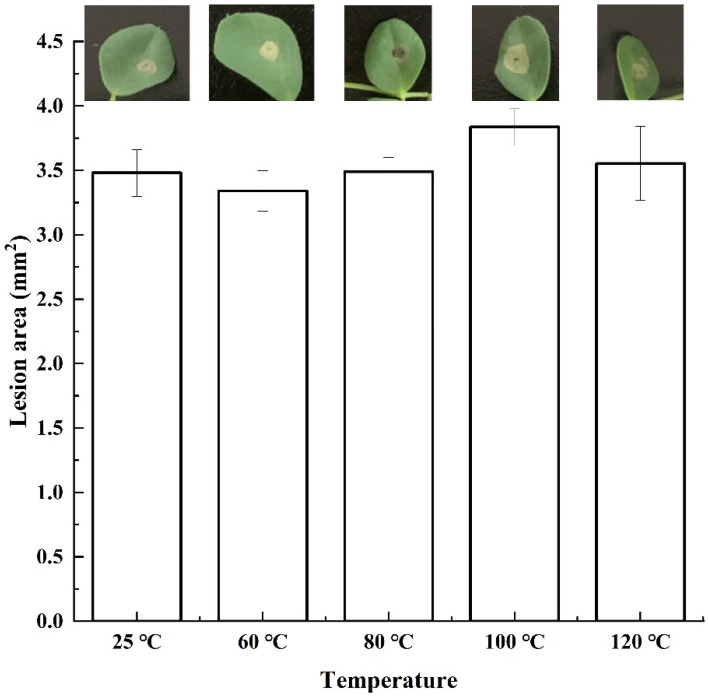
The phytotoxicity of crude toxin after treated by different temperature. The mean lesion area was calculated from six leaves of tissue-cultured alfalfa plantlets (*n* = 6). Values (means + SE) marked with different letters in each column indicate significant differences (*p* < 0.05) based on one-way ANOVA followed by Duncan’s multiple range test.

Crude toxin caused lesions with slightly different areas after treatment with different temperatures (Figure 3). The crude toxin caused a lesion area of 3.6 mm^2^ after incubation at 120 °C, indicating that the crude toxin had a highly thermostable toxic activity. 

### 3.3. Effect of the Crude Toxin on Susceptible and Resistant Cultivars

#### 3.3.1. Effect of Crude Toxin in Water Medium

**Figure 4:**
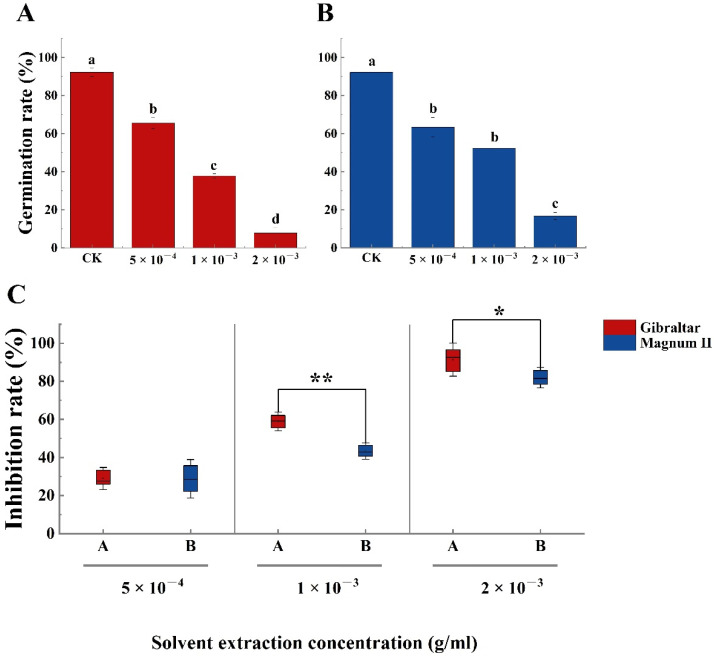
The germination rate of susceptible and resistant cultivars seeds treated with different concentrations of crude toxin in water medium. (**A**) crude toxin effect on germination rate of susceptible cultivar. (**B**) crude toxin effect on germination rate of resistant cultivar. (**C**) the different inhibition rates of germination between the susceptible and the resistant cultivars. The test of germination rate was calculated from 90 (*n* = 90). Values (means + SE) marked with different letters in each column indicate significant differences (*p* < 0.05) based on one-way ANOVA followed by Duncan’s multiple range test. The significant different inhibition rate between susceptible and resistant cultivars, * indicate *p* < 0.05, ** indicate *p* < 0.01.

The phytotoxin effect of the crude toxin was examined using susceptible (Gibraltar) and resistant (Magnum II) alfalfa cultivar seeds. Compared with the negative control, the germination rates of susceptible and resistant cultivar seeds were significantly different at various concentrations of crude toxin (Figure 4A,B). The germination rate decreased with the increasing concentration of crude toxin. The germination rates of susceptible (65.6%) and resistant (63.3%) cultivars were significantly lower than that of the control at a crude toxin concentration of 5 × 10^−4^ g/mL. The germination rates of susceptible and resistant cultivars were 37% and 52%, respectively, when the concentration of crude toxin was 1 × 10^−3^ g/mL. When the crude toxin concentration was 2 × 10^−3^ g/mL, the germination rate of susceptible and resistant cultivars was 7.8% and 16.7%, respectively. Furthermore, seed germination inhibition rates of susceptible (28.9%) and resistant (28.8%) cultivars were not significantly different at crude toxin concentrations of 5 × 10^−4^ g/mL. The seed germination inhibition rate increased with increasing concentrations of crude toxin. The difference between the susceptible and the resistant cultivars was significant. The seed germination inhibition rate of the susceptible cultivar (59%) was significantly higher than the resistant cultivar (43.3%) (*p* < 0.01) at the concentration of 1 × 10^−3^ g/mL. At the 2 × 10^−3^ g/concentration, the inhibition rate of the susceptible cultivar (91.4% was still significantly higher than the resistant cultivar (81.9%) (*p* < 0.05) (Figure 4C).

**Figure 5:**
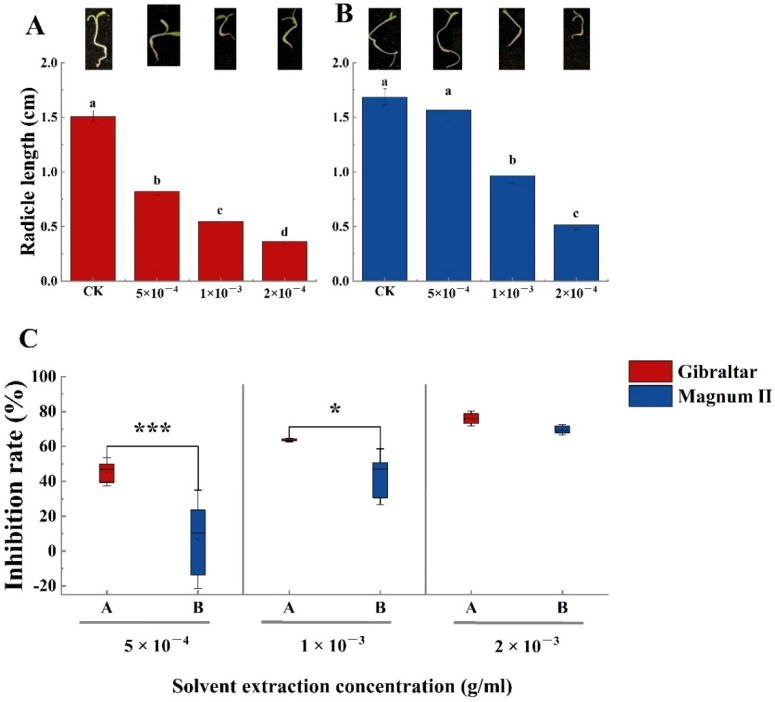
The radicle length of susceptible and resistant cultivars treated with different concentration of crude toxin in water medium. (**A**) crude toxin effect on radicle length of susceptible cultivar. (**B**) crude toxin effect on radicle length of resistant cultivar. (**C**) difference in inhibition rates of radicle length between the susceptible and resistant cultivars. The test of radicle length was calculated from 45 (*n* = 45). Values (means + SE) marked with different letters in each column indicate significant differences (*p* < 0.05) based on one-way ANOVA followed by Duncan’s multiple range test. The significant different inhibition rate between susceptible and resistant cultivars, * indicate *p* < 0.05, *** indicate *p* < 0.001.

Meanwhile, all crude toxin concentrations significantly affected the radicle length of the susceptible cultivar (Figure 5A). The average radicle root length in the negative control was 1.5 cm after inoculation for 3 days, which was significantly longer than that of plants treated with the crude toxin. The radicle length was 0.8, 0.54, and 0.36 cm at crude toxin concentrations of 5 × 10^−4^, 1 × 10^−3^, 2 × 10^−3^ g/mL, respectively. Moreover, the radicle length inhibition rate of the susceptible cultivar reached 44.5% at a crude toxin concentration of 5 × 10^−4^ g/mL. The inhibition rate increased to 62.6 and 75.8% when the crude toxin concentrations were 1 × 10^−3^ and 2 × 10^−3^ g/mL, respectively (Figure 5C). The crude toxin concentration of 5 × 10^−4^ g/mL did not significantly affect the root length (1.56 cm) of the resistant cultivar compared with the negative control (1.7 cm). However, the root length significantly decreased to 0.96 and 0.51 cm at crude toxin concentrations of 1 × 10^−3^ and 2 × 10^−3^ g/mL, respectively. Furthermore, the root length inhibition rates of the resistant cultivar were significantly different at different crude toxin concentrations. The root length inhibition rate was only 4.8% when the crude toxin concentration was 5 × 10^−4^ g/mL. However, the root length inhibition rate increased to 41.7 and 68.6% at crude toxin concentrations of 1 × 10^−3^ and 2 × 10^−3^ g/mL, respectively. Besides, the root length inhibition rate was significantly different (*p* < 0.001 and *p* < 0.05) between the susceptible and resistant cultivars when the crude toxin concentrations were 5 × 10^−4^ and 1 × 10^−3^ g/mL (Figure 5C). The radicle length inhibition rate was not significantly different between the susceptible and resistant cultivars at crude toxin concentration of 2 × 10^−3^ g/mL (Figure 5C). 

#### 3.3.2. Effect of Crude Toxin in MS Medium

**Figure 6:**
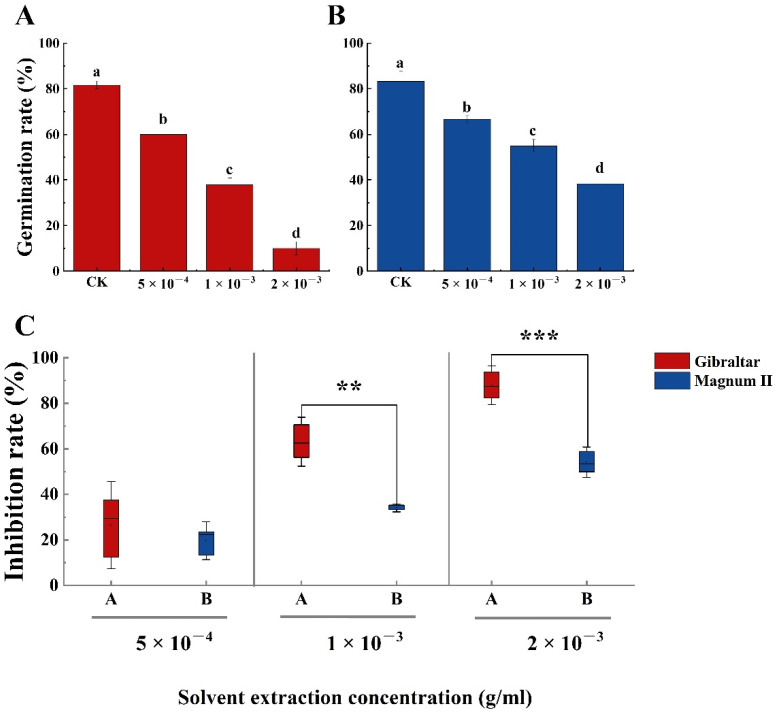
The germination rate of susceptible and resistant cultivars seeds treated with different concentrations of crude toxin in MS medium. (**A**) crude toxin effect on germination rate of susceptible cultivar. (**B**) crude toxin effect on germination rate of resistant cultivar. (**C**) difference in inhibition rate of germination between the susceptible and the resistant cultivars. The test of germination rate was calculated from 60 (*n* = 60). Values (means + SE) marked with different letters in each column indicate significant differences (*p* < 0.05) based on one-way ANOVA followed by Duncan’s multiple range test. The significant different inhibition rate between susceptible and resistant cultivars, ** indicate *p* < 0.01, *** indicate *p* < 0.001.

Compared with the control, seed germination rates of susceptible and resistant cultivars significantly decreased in MS medium with increasing crude toxin concentration (Figure 6A,B). The germination rates of the susceptible cultivar were 45, 30, and 10% when the concentrations of the crude toxin were 5 × 10^−4^, 1 × 10^−3^, and 2 × 10^−3^ g/mL, respectively, while the germination rates of the resistant cultivar were 66, 55, and 38%, respectively. Moreover, the seed germination inhibition rates were significantly different between susceptible and resistant cultivars at each crude toxin concentration (*p* < 0.001) (Figure 6C). The seed germination inhibition rates of susceptible and resistant cultivars were 44.7% and 19.7%, respectively, at a crude toxin concentration of 5 × 10^−4^ g/mL; 63.1% and 34.0%, respectively, at a crude toxin concentration of 1 × 10^−3^ g/mL; and 87.9 and 54.1%, respectively, at a crude toxin concentration of 2 × 10^−3^ g/mL. 

**Figure 7:**
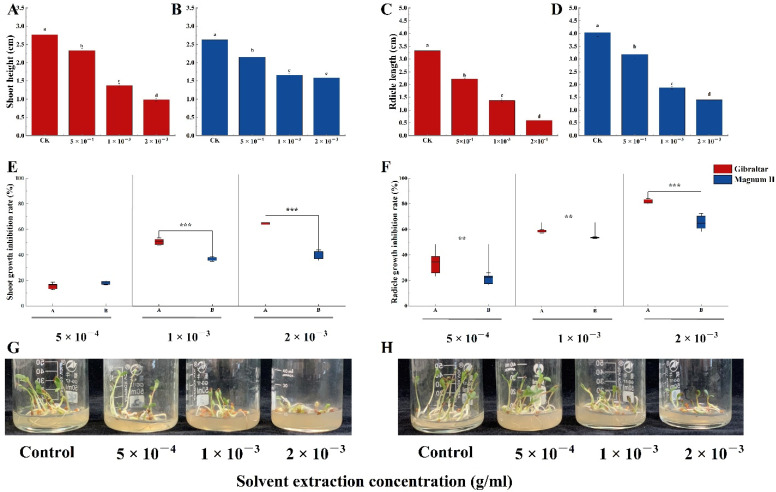
The shoot and radicle length of susceptible and resistant cultivars treated with different concentrations of crude toxin in MS medium. (**A**) crude toxin effect on shoot length of susceptible cultivar. (**B**) crude toxin effect on shoot length of resistant cultivar. (**C**) crude toxin effect on radicle length of susceptible cultivar. (**D**) crude toxin effect on radicle length of resistant cultivar. (**E**) the difference in inhibition rates of shoot length between the susceptible and the resistant cultivars. (**F**) the difference in inhibition rates of radicle length between the susceptible and the resistant cultivars. Photograph of the experimental set up clearly exhibiting the inhibitory effect of different concentrations of crude toxin on the pre-germinated alfalfa seedling growth of susceptible (**G**) and resistant (**H**) cultivars. The test of shoot and radicle length was calculated from 45 (*n* = 45). Values (means + SE) marked with different letters in each column indicate significant differences (*p* < 0.05) based on one-way ANOVA followed by Duncan’s multiple range test. The significant different inhibition rate between susceptible and resistant cultivars, ** indicate *p* < 0.01, *** indicate *p* < 0.001.

Furthermore, the crude toxin significantly affected the shoot and radicle growth of the two cultivars (Figure 7G,H). Compared with the control (2.76 cm), crude toxin significantly decreased the shoot lengths of the susceptible cultivar to 2.3, 1.4, and 1.0 cm at concentrations of 5 × 10^−4^, 1 × 10^−3^, and 2 × 10^−3^ g/mL, respectively (Figure 7A). Compared with the control (3.3 cm), crude toxin significantly decreased the radicle length of the susceptible cultivar to 2.2, 1.4, and 0.6 cm at 5 × 10^−4^, 1 × 10^−3^, and 2 × 10^−3^ g/mL, respectively (Figure 7C). For the resistant cultivar, crude toxin significantly reduced the shoot and radicle lengths compared with the control (shoot length; 2.6 cm and radicle length; 4.0 cm) (Figure 7B,D). The shoot lengths were 2.2, 1.7, and 1.6 cm at crude toxin concentrations of 5 × 10^−4^, 1 × 10^−3^ and 2 × 10^−3^ g/mL, respectively, and radicle lengths were 3.2, 1.9, and 1.4 cm, respectively. Moreover, the shoot length inhibition rates were not significantly different between the susceptible (15.5%) and the resistant (18.0%) cultivars at crude toxin concentration of 5 × 10^−4^ g/mL (Figure 7E). The shoot length inhibition rates were significantly different between the susceptible cultivar (50.3%) and the resistant cultivar (36.9%) at a crude toxin concentration of 1 × 10^−3^ g/mL (*p* < 0.001). Similarly, the shoot length inhibition rate was significantly higher in susceptible cultivar than in resistant cultivar (64.3% vs. 50.3%) at crude toxin concentration of 2 × 10^−3^ g/mL (*p* < 0.001). Furthermore, the radicle length inhibition rates significantly differed between susceptible and resistant cultivars at various crude toxin concentrations (Figure 7F). The radicle length inhibition rates at crude toxin concentrations of 5 × 10^−4^, 1 × 10^−3^, and 2 × 10^−3^ g/mL were 32.9, 58.6, and 81.7%, respectively, for the susceptible cultivar and 21.0, 53.5, and 65.2%, respectively, for the resistant cultivar. 

### 3.4. Transmission Electron Microscopy

**Figure 8:**
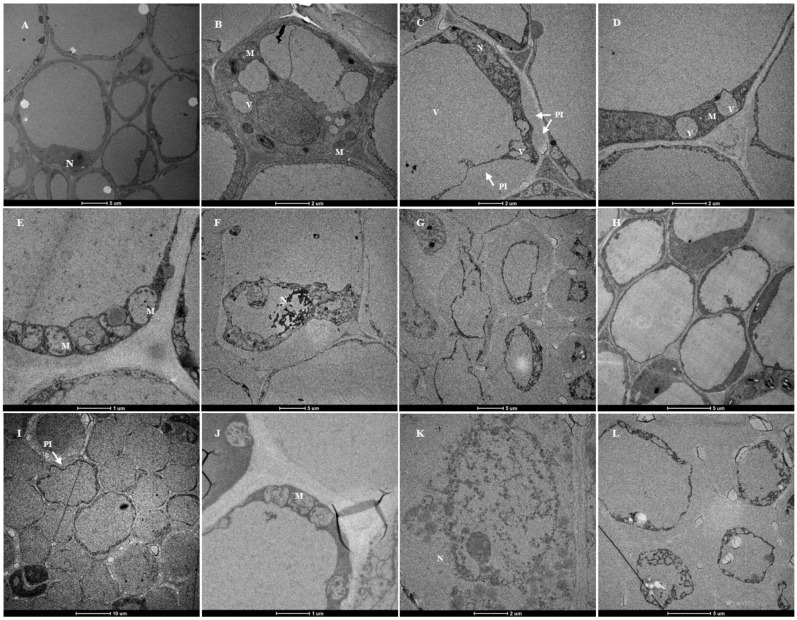
Ultrastructure of root cortical cells of alfalfa susceptible and resistant cultivars treated with crude toxin (2 × 10^−3^ g/mL). (**A**,**B**) ultrastructure of root cortical cells of susceptible cultivar treated with 20% ethyl acetate for 24 h (N, nuclear. M, mitochondria). (**C**,**D**) plasma-membrane invaginations (PI) and irregular shape small vacuoles (V) in susceptible cultivar root treated with crude toxin for 3 h. (**E**) deformation of mitochondria with loss of cristae in susceptible cultivar root treated with crude toxin for 6 h. (**F**) the nuclear membranes were disrupted in susceptible cultivar root treated with crude toxin for 12 h. (**G**) severe plasmolysis of cells in susceptible cultivar root treated with toxin for 12 h. (**H**) ultrastructure of root cortical cells of resistant cultivar treated with 20% ethyl acetate for 24 h. (**I**) observed plasma membrane in a few cells in resistant cultivar root treated with crude toxin for 6 h. (**J**) deformation of mitochondria with loss of cristae in susceptible cultivar root treated with crude toxin for 12 h. (**K**) nuclear membrane in resistant cultivar root treated with crude toxin for 12 h. (**L**) the plasma membrane in resistant cultivar was exacerbated after treatment with crude toxin for 24 h.

Ultrastructural studies showed that the root cortical cells of plants treated with a crude toxin concentration of 2 × 10^−3^ g/mL and the control plants (grown in 20% ethyl acetate for 24 h) were significantly different (Figure 8). The cortical cells of the control plants had a typical ultrastructure with intact cell walls and large vacuoles. The dense cytoplasm was located at a relatively narrow peripheral position in the cell. The cytoplasm also contained compact mitochondria, nuclei with nucleolus, and normal-looking small vacuoles (Figure 8A,B,H).

However, the susceptible cultivar had abnormal cortical cell structure after crude toxin treatment. Ultrastructural changes in plasma membrane and small vacuoles first occurred after 3 h of crude toxin treatment. The cortical cells had invaginated plasma membrane. The membrane of small vacuoles had an irregular shape (Figure 8C,D). Moreover, mitochondria were deformed with loss of cristae after 6 h of crude toxin treatment (Figure 8E). The nuclear membranes were disrupted, and the plasmolysis was aggravated after 12 h of crude toxin treatment (Figure 8F,G). 

For resistant cultivar, invagination of plasma membrane occurred in a few root cortical cells after 6 h of crude toxin treatment (Figure 8I). The mitochondria were deformed, and some mitochondrial cristae disappeared after 12 h of crude toxin treatment (Figure 8J). Crude toxin did not affect the nuclear envelope in root cortical cells for the first 12 h (Figure 8K). However, the plasma membrane in cortical cells was significantly invaginated after 24 h of crude toxin treatment (Figure 8L). 

### 3.5. Host Selectivity Test

**Figure 9:**
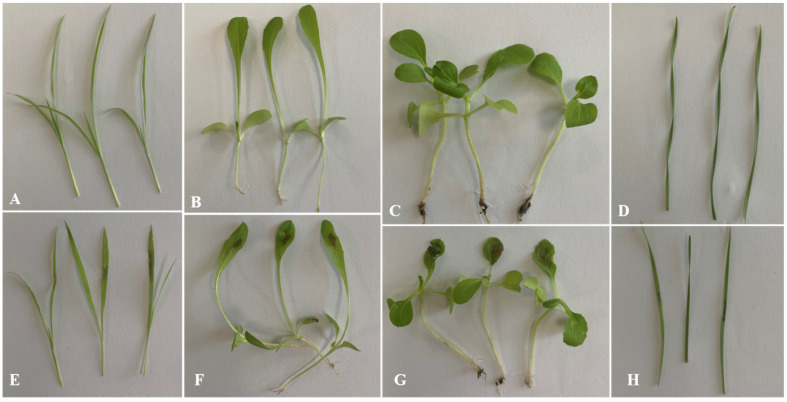
Phytotoxin of crude toxin on the leaves of *Sonchus oleraceus* (**E**), *Capsellabursa-pastoris* (**F**), *Chenopodium album* (**G**), and *Agropyron cristatum* (**H**) changed after treated for 72 h. (**A**) control of *Sonchus oleraceus*. (**B**) control of *Capsellabursa-pastoris*. (**C**) control of *Chenopodium album*. (**D**) control of *Chenopodium album*.

Lesions developed on the leaves of *Sonchus oleraceus* L., *Capsella bursa-pastoris* (Linn.) Medic, *Chenopodium album* L., and *Agropyron cristatum* L. after inoculation for 3 days (Figure 9). The lesion area of *Sonchus oleraceus* leaves was chlorotic (Figure 9E), while the lesion area of *Capsellabursa-pastoris* leaves turned brown, and the adjacent tissues were curled (Figure 7F). Crude toxin caused the *Chenopodium album* leaf tissue to sink, and the lesions were brown, watery, and rotten (Figure 9G). The *Agropyron cristatum* leaves were discolored at the edge near the inoculation site (Figure 9H).

**Figure 10:**
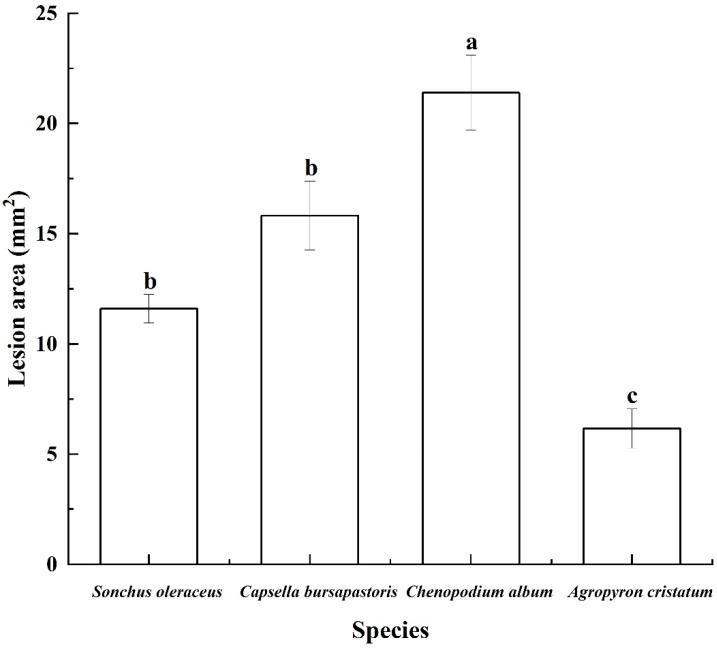
The lesion area caused by crude toxin on *Sonchus oleraceus*, *Capsellabursa-pastoris*, *Chenopodium album*, and *Agropyron cristatum* leaves. Phytotoxic test was carried out with a concentration of 2 × 10^−3^ g/mL. The mean lesion area was calculated from three leaves (*n* = 3). Values (means + SE) marked with different letters in each column indicate significant differences (*p* < 0.05) based on one-way ANOVA followed by Duncan’s multiple range test.

The lesion areas on *Chenopodium album*, *Sonchus oleraceus*, *Capsellabursa-pastoris*, and *Agropyron cristatum* leaves were 21.4 mm^2^, 11.6 mm^2,^ 15.8 mm^2^, and 6.1 mm^2^, respectively (Figure 10).

### 3.6. Gas Chromatography-Mass Spectrometry

Gas chromatography-mass spectrometry (GC-MS) analysis after ethyl acetate fractionation showed many peaks. Compounds with similarities of less than 70% were filtered out. The few compounds were selected based on the unique nature and relative abundance of the peaks. Finally, four compounds were detected using GC-MS analysis: 3-hydroxypyridine, 5-methylresorcinol, 3-Hydroxypropionic acid, and 4-Hydroxyphenylethanol (Table 1).

## 4. Discussion

This study is the first to investigate the elementary properties of crude toxin extracted from *P. radicina*, its activity, and the influence on susceptible and resistant cultivars. The following outcomes were found in this study: (i) the crude toxin of *P. radicina* has different effects on seed germination, shoot length, and radicle length of susceptible and resistant cultivars; (ii) the crude toxin affected the root cortical cell plasma membrane and cell wall, and disrupted the structure of cellular organelles; (iii) the crude toxin is also phytotoxic to *S. oleraceus*, *C. pastoris*, *C. album*, and *A. cristatum*; and (iv) GC-MS analysis identified four compounds in the crude toxin. 

According to the two-way ANOVA analysis, the filtrate from MEB culture for 14 days caused the largest lesion area on alfalfa leaves. Several studies have found that the phytotoxicity of secondary metabolites obtained after different durations of fermentation and exposure times are significantly different. For instance, *Ascochyta rabiei* toxin from Czapek-Dox medium with chickpea seed extract can kill the cells of chickpea leaflets because the host plants contain a toxin inducing substance [31]. The culture filtrate of *Stemphylium solani* from PSB has a larger lesion area than other medium. However, its toxicity reduces when incubated for more than 30 days [32]. Filtrates from *Lasiodiplodia theobromae* have the highest phytotoxicity after 21 days in liquid cultures of Richard [33]. Herein, phytotoxicity was reduced at 21 days compared with at 14 days, possibly due to the decreased medium nutrition available for pathogen or toxin in culture medium degradation. Therefore, it is necessary to select the toxin production culture medium and the incubation time.

Solvent extraction to recover crude toxin compounds with novel activity is one of the most effective methods to isolate phytotoxins from pathogens [34]. Herein, toxic compounds extracted using ethyl acetate had a higher phytotoxicity than those extracted using other organic solvents. A previous study showed that ethyl acetate could effectively extract substances with a significantly higher toxicity from culture filtrate than other organic solvents [27,35]. Our research also confirmed that the phytotoxin was stable even at 120 °C and had high toxicity, indicating that it may neither be an enzyme nor a protein

Furthermore, the activity of phytotoxins was assessed based on seed germination, shoot, and radicle growth of susceptible and resistant cultivars. The inhibition rates increased with increasing crude toxin concentrations. The tolerance of resistant cultivars to the crude toxin was significantly higher than that of the susceptible cultivar based on germination, shoot length, and radicle length inhibition rates. A previous study also showed that resistant cultivars could tolerate a significantly higher concentration of secondary metabolites of *Fusarium* than the susceptible cultivar [36]. As a result, several studies have researched the relationship between toxin reaction and plant resistance. For instance, a study used phytotoxin from *Rhizoctonia solani* to assess 17 resistant rice cultivars and found a significant positive correlation between toxin sensitivity and disease susceptibility [37]. Moreover, identifying resistance to phytotoxin thaxtomin from Streptomyces is necessary for developing resistant potato cultivars because the phytotoxin is capable of inducing common scab disease symptoms without pathogen strains [38,39]. Culture filtrate has also been used to differentiate banana cultivars resistant to *Fusarium oxysporum* f. sp. in field growth [17,40]. Herein, the crude toxin in MS medium significantly affected plant and root growth. Similarly, a glasshouse experiment showed that *P. radicina* root dip inoculation reduces the aboveground and belowground alfalfa tissues [1]. Although a significant difference has been observed between the susceptible and resistant cultivars after treatment with crude toxin, additional research is needed to determine the contribution of the toxin to pathogen virulence.

TEM studies showed that the ultrastructural characteristics of root cortical cells of susceptible and resistant cultivars were different after crude toxin treatment. The crude toxin significantly affected the root cortical cells of the susceptible cultivar and much faster than those of resistant cultivars. For instance, the first observable crude toxin induced changes in susceptible cultivar appeared on the plasma membrane cell wall and the irregular shape of small vacuoles after 3 h. The mitochondria and nuclear membrane structures were damaged as treatment time increased. A previous study showed that phytotoxin has three different targets in host cells: plasma membrane, chloroplasts, and mitochondria [41]. A study showed that AK-toxin I from *Alternate* can damage the plasma membrane cell of Japanese pear leaves, characterized by invagination of the plasmalemma [42]. Moreover, the host treated with the phytotoxin can cause mitochondria swelling, the disappearance of matrix components, and cristae destruction [43]. The susceptible cultivar leaves of garlic exposed to SS-toxin from *Stemphylium solani* have a reduced number of mitochondrial cristae [44]. In our previous study, when *P. radicina* infected susceptible and resistant cultivars, the differences in germination rate, hyphal growth, and infection rate were significant in the early stage (unpublished). These results suggest that crude toxin might be involved in the early stage of the infection. The *P. radicina* phytotoxin causes partial dysfunction of target structures, resulting in a partial disturbance of the membranes or the metabolism of organelles, thus enhancing pathogen infection. The resistant cultivar may contain inhibitors in cortical cells, thus inhibiting phytotoxin migration in plant cells. 

The crude toxin is non-host selective since it can cause a lesion in other plant leaves. Herein, the crude toxin induced more lesions in *S. oleraceus*, *C. pastoris*, and *C. album* leaves than in alfalfa leaves. It could be due to two reasons. First, the epicuticular wax of alfalfa leaves could be thicker than that of *Sonchus oleraceus* L., *Capsella bursa-pastoris* (Linn.) Medic, and *Chenopodium album* L. *Agropyron cristatum* L. leaves. Second, on alfalfa leaves treated with crude toxin, we could observe the obvious chlorosis and the distinct lesion border. This could be the hypersensitive necrosis reaction of the plant. However, on the *Capsella bursa-pastoris* (Linn.) Medic leaves around the lesion area we observed a yellow halo. The lesion border was not clear on both *Sonchus oleraceus* L. and *Chenopodium album* L. leaves. A previous study showed that *S. oleraceus*, *C. pastoris*, *C. album*, and *A. cristatum* are the common weeds in the alfalfa planting area [45]. Weed invasion significantly affects production and the quality of alfalfa [46,47], especially during the establishment period of alfalfa seedlings [48]. Currently, chemical herbicides are widely used to control weeds. However, phytotoxin provides a new method for developing an ecofriendly alternative for weed control. Besides, some phytotoxins have been patented [19]. Phytotoxin produced by numerous *Phoma*-like fungi can be used to control weeds. For instance, *Phoma exigua* is a potential biocontrol agent of various weeds, such as *Taraxacum officinalis*, *Gaultheria shallon,* and *Cirsium arvense* [35]. Two substances, macrocidin A and B, with bleaching and chlorosis effect on broadleaf, have been isolated from *Phoma macrostoma* [49]. In this study, the crude toxin showed phytotoxin activity on leaves of four weeds, especially *C. album*, which had decayed lesions. The lesion area was larger in *Sonchus oleraceus*, *Capsellabursa-pastoris,* and *Chenopodium album* leaves than in alfalfa leaves. These results suggest that the phytotoxins from *P. radicina* have herbicidal effects.

Moreover, GC-MS analysis identified four compounds. These compounds have been isolated from fungi, with some having phytotoxic effects on plants. For instance, 4-Hydroxyphenylethanol has been isolated from *Leptographium qinglingensis* carried by *Dendroctonus armandi*, however, it does not induce phytotoxicity in pine needles [50]; 5-methylresorcinol has been isolated from a culture of *Paecilomyces verticillatus* [51]; 3-Hydroxypyridine has been isolated from a culture of *Fusarium moniliforme* [52]; and 3-Hydroxypropionic acid has been isolated from several endophytic fungi, and it has phytotoxic effects on plant-parasitic nematode. Moreover, it affects *Setaria italica* at 600 μg/mL [17]. A proper understanding of the chemistry compound and its role in pathogenesis requires further investigation and current investigations provide a proper base for this.

To the best of our knowledge, this is the first study to report crude toxin from *P. radicina*. In this study, the optimal medium, time, and extraction solvent were determined. Compared with the resistant cultivar, the susceptible cultivar was vulnerable to the crude toxin. Furthermore, ultrastructure studies showed that crude toxin caused plasmolysis and organelle collapse. The crude toxin is non-host selective, indicating that it has the potential for herbicidal effects. GC-MS analysis selected four compounds for the follow-up experiments. Therefore, this study provides a basis for understanding the pathogenic mechanisms of *P. radicina* on alfalfa roots.

## Figures and Tables

**Table 1:** Toxic compounds identified from crude toxin through GC-MS analysis.

S. No.	RT	Name of the Compound	Mol. Formula	MW	Peak Area	CAS	Structure
1	14.35	4-Hydroxyphenylethanol	C8H10O2	179	3.50%	2380-91-8	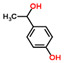
2	12.79	5-methylresorcinol	C7H8O2	253	1%	504-15-4	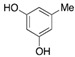
3	8.61	3-hydroxypyridine	C5H5NO	152	0.90%	109-00-2	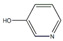
4	14.38	3-Hydroxypropionic acid	C3H6O3	103	2.80%	503-66-2	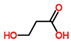

RT: Retention Time; MW: Molecular Weight; CAS: Chemical Abstracts Service.

## Data Availability

Data are contained within the article.
